# Evaluating the clinical efficacy and safety of concurrent chemoradiotherapy with cisplatin and nab-paclitaxel in postoperative early-stage cervical cancer

**DOI:** 10.1007/s00432-024-05764-9

**Published:** 2024-05-06

**Authors:** Ning Zhao, Yunhai Li, Xue Chen, Jinli Ma, Weiming Luo, Yunhai Li

**Affiliations:** 1 Department of Radiation Oncology, Minhang Branch Hospital, Fudan University Shanghai Cancer Center, Shanghai 200240, China; 2 Department of Radiation Oncology, Fudan University Shanghai Cancer Center, Fudan University, Shanghai 200032, China

**Keywords:** Cervical cancer, Concurrent chemoradiotherapy, Nab-paclitaxel, Cisplatin, Toxicity, Survival

## Abstract

**Objective:**

A preclinical study showed that nab-paclitaxel acted as a radiosensitizer and improved tumor radiotherapy in a supra-additive manner. In this study, we aimed to evaluate the clinical efficacy and safety of concurrent chemoradiotherapy (CCRT) with cisplatin and nab-paclitaxel in postoperative early-stage cervical cancer with an unfavorable prognosis.

**Methods:**

Eligible patients with stage IB1-IIA2 (FIGO 2009) cervical carcinoma were recruited retrospectively between August 2018 to May 2021. Patients in both the cisplatin and nab-paclitaxel groups received postoperative radiotherapy and weekly intravenous cisplatin 40 mg/m^2^ or nab-paclitaxel 100 mg concurrently. An analysis of overall survival, progression-free survival, and adverse reactions was conducted.

**Results:**

A total of 105 early-stage cervical cancer patients were included into our study. The median follow-up time was 38.7 months. The 3-year overall survival and progression-free survival in both group was similar. The cycles of chemotherapy in the cisplatin group were less than those in the nab-paclitaxel group (4.5 vs. 5.0; p = 0.001). Patients in the cisplatin group had a significantly higher frequency of hematological adverse events than patients in the nab-paclitaxel group (P < 0.05). Patients in the cisplatin group had a significantly higher frequency of grade 3–4 leukopenia (46.1% vs. 18.9%; P = 0.03), grade 1–2 thrombocytopenia (32.7% vs. 9.5%; P = 0.014) than patients in the nab-paclitaxel group. Gastrointestinal reactions, such as vomiting, nausea, and anorexia were significantly reduced in the nab-paclitaxel group compared with those in the cisplatin group. Regarding the effects on alopecia, the incidence rate of the nab-paclitaxel group was higher than that of the cisplatin group (P = 0.001). There were no differences between the groups in terms of other adverse reactions.

**Conclusion:**

The results of this study indicate that nab-paclitaxel-based concurrent radiotherapy is tolerable and effective, and can be considered an alternative to cisplatin chemotherapy.

## Introduction

Cervical cancer is the fourth most common cancer in the world for female malignancies, and a fully preventable disease, but remains the main cause of cancer death in women in 36 low-income and middle-income countries (LMICs) (Sung et al. 2021; Arbyn et al. 2020). For early-stage cervical cancer (IB1-IIA2), radical surgery remains the preferred choice in clinical practice. Based on postoperative pathological diagnosis, adjuvant radiotherapy with concurrent cisplatin or cisplatin-based chemotherapy has been advocated for postoperative patients with pathological high-risk factors, such as positive vaginal resection margin, lymph node metastasis, parametrial invasion (Peters et al. 2023; Okazawa et al. 2013). Additionally, the National Comprehensive Cancer Network (NCCN) guidelines mentioned that concurrent platinum and radiotherapy (category 2B) is proposed for some patients presenting with intermediate-risk factors, such as large tumor size (> 4 cm), deep (> 1/3) stromal invasion, or lymphatic vascular space involvement (LVSI) (Okazawa et al. 2013). Cisplatin, remains a first-line treatment agent that has predominated the chemotherapy of cervical cancer for a long time in clinical practice. As we know, cisplatin may cause severe gastrointestinal reactions and nephrotoxicity and cannot be tolerated by some patients (Li et al. 2022b, a). As a result, developing new alternative formulation is imperative. Nab-paclitaxel, a novel, chremophor-free 130-nm nanoparticle albumin-bound formulation, paclitaxel was transported and concentrated in cancer cells through serum albumin, which binds a specific receptor (gp60) on the endothelia, function as a carrier (Minshall et al.^2002^). This preparation improved pharmacokinetic and pharmaco-dynamic properties, including the needless of antiallergic premedication, faster tumor penetration, and increased antitumor activity as compared to standard paclitaxel (Gardner et al. 2008; Desai et al. 2006). Although it has been used as a concurrent and neoadjuvant CT agent for locally advanced cervical cancer and showed strong antitumor efficacy (Mandloi et al. 2019; Li et al. 2021). Reports to date regarding the use of nab-paclitaxel in the treatment of patients with early-stage cervical cancer appear to be infrequent. Accordingly, we conducted this retrospective control study to assess the clinical efficacy and adverse reactions of nab-paclitaxel- or cisplatin based concurrent chemoradiotherapy (CCRT) in postoperative early-stage cervical cancer with an unfavourable prognosis.

## Methods

### Patients and study design

Eligible cervical carcinoma patients underwent radical hysterectomy and pelvic lymph node dissection and stage IB1-IIA2 according to the International Federation of Gynecology and Obstetrics (FIGO 2009) between August 2018 to May 2021 at our institution. These patients were between the ages of 22 and 73 years with histologically confirmed cervical cancer (including squamous cell carcinoma, adenocarcinoma, mixed adenosquamous carcinoma) and were pathologically confirmed to have at least one of the following adverse factors confirmed by postoperative pathological examination: lymph node metastasis, positive parametrium or margins, lymphatic vascular space involvement, or deep stromal invasion. For these intermediate-risk patients, concurrent chemoradiotherapy was recommended at our institution. Exclusion criteria included patient’s request for exit, serious adverse event; progressive disease (PD) and poor compliance. All patients needed to accept a pre-treatment evaluation, including provide medical history, blood routine examination, biochemical laboratory tests, abdominal and gynecological examination, pelvic magnetic resonance imaging (MRI), thorax and abdomen computed tomography(CT) or whole-body positron emission tomography (PET)-CT.

This study was approved by our institutional medical ethics committee (2021006). The decision to recommend concurrent chemoradiotherapy was at physician and patient discretion. All participants consented to the use of their medical records for research purposes.

### Procedures and treatments

Both the groups received concurrent radiochemotherapy within 4–6 weeks after surgery. Pelvic external beam radiation therapy (EBRT) in this study was carried out using 6MV photons in a linear accelerator (1.8–2.1 Gy/fraction/day, 5 days/week, 25–28 fractions for a total dose of 45–58.8 Gray). Patients in the cisplatin group were given cisplatin dose of 40 mg/m^2^, and nab-paclitaxel was administered at a dose of 100mg intravenously over 30 min for patients in the nab-paclitaxel group, for a maximum of 6 doses during radiation. During the administration of nab-paclitaxel, a cardiac monitor was applied and blood pressure, heart rate, and respiration rate were recorded every 15 min. The two regimes were used weekly for four to six doses until the end of radiotherapy, serious adverse reactions, or other personal reasons. During treatment, blood samples were taken twice a week before and after chemotherapy to evaluate the blood count and renal and liver functions were also been monitored every two weeks, based on the results patients would be given symptomatic treatment. Other adverse effects such as gastrointestinal reactions, alopecia and neurotoxicity also were recorded.

### Evaluation and follow-up of therapeutic interventions

Treatment efficacy of patients was evaluated every 3 months for the first 2 years, every 6 months during the next 3 years and thereafter annually, with gynaecological examination, serum tumor markers and imaging tests. All patients were followed up until death or 28 February 2023. The primary endpoint of the study was therapy-associated toxicity, the secondary endpoints were progression-free survival (PFS) and overall survival (OS) at 3 years. PFS was defined as the time from study enrollment to first documented disease progression or death from any cause. OS was the time between randomization and death or censoring at the date of the last follow-up.

### Statistical analysis

Statistical analyses were performed by SPSS 22.0 software. Categorical variables were assessed using the *χ*^2^ test and Fisher's-exact test, while continuous variables were evaluated using the Mann–Whitney *U* test. The *χ*^2^ test was employed to compare adverse events. Survival outcomes were estimated using the Kaplan–Meier method and assessed using the log-rank test. Statistical significance was determined at a threshold of P < 0.05.

## Results

### Baseline characteristics

**Fig. 1:**
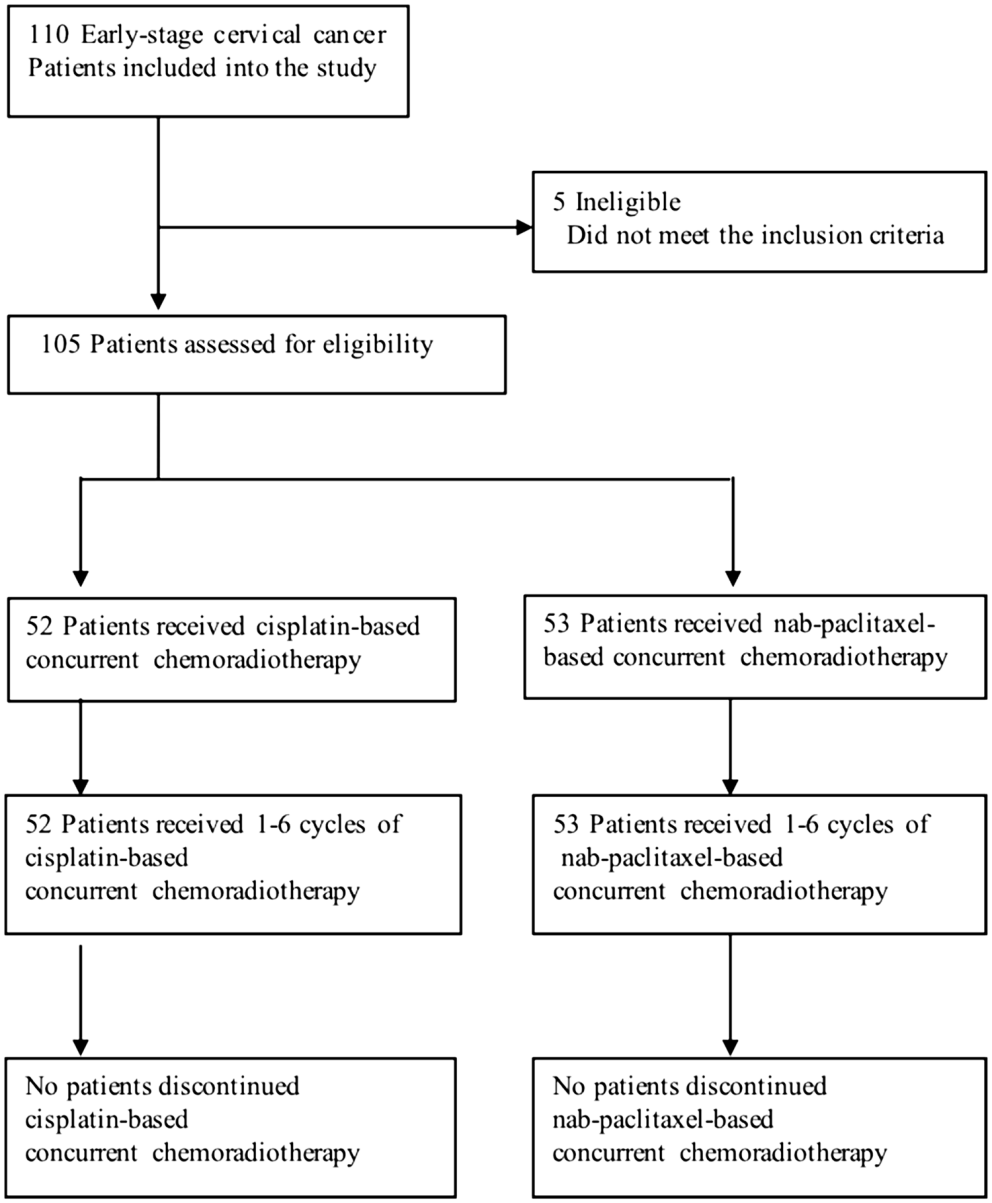
The enrollment and treatment of patients in this study

Between August 2018 to May 2021, 110 early-stage cervical cancer patients were included into the study. A total of 3 patients in the cisplatin group and 2 patients in the nab-paclitaxel group were excluded from the trial on the basis of the inclusion and exclusion criteria. 52 patients completed a 1–6 cycles of cisplatin-based CCRT and 53 patients completed a 1–6 cycles of nab-paclitaxel-based CCRT (Fig. 1). Baseline demographic and clinical characteristics are summarized in Table 1. No differences were observed in parameters in two treatment groups, other than the cycles of chemotherapy. The cycles of chemotherapy in the cisplatin group were less than those in the nab-paclitaxel group (4.5 vs. 5.0; P = 0.001).

**Table 1:** Patient characteristics

	Cisplatin group(N = 52)	Nab-paclitaxel group (N = 53)	p value
*Age*			
Median (years)	50	52	0.177
Range (years)	27–73	22–68	
*Histological pattern*			
Squamous cell carcinoma	44 (84.6)	45 (84.9)	0.88
Adenocarcinoma	4 (7.7)	5 (9.4)	
Adenosquamous carcinoma	4 (7.7)	3 (5.7)	
*FIGO stage*			
IB1	3 (5.8)	7 (13.2)	0.505
IB2	17 (32.7)	14 (26.4)17	
IB3	10 (19.2)	6 (11.3)	
IIA1	6 (11.5)	6 (11.3)	
IIA2	16 (30.8)	20 (37.7)	
*Cycles of chemotherapy*			
3	6 (11.5)	1 (1.9)	0.001*
4	14 (26.9)	10 (18.9)	
5	31 (59.6)	28 (52.8)	
6	1 (1.9)	14 (26.4)	
*Dose*			
45 Gy/25Fx	24 (46.2)	17 (32.1)	0.234
50.4 Gy/28Fx	17 (32.7)	18 (33.9)	
58.8 Gy/28Fx	11 (21.1)	18 (33.9)	

Data are shown as n (%), unless otherwise specified

*FIGO*, Federation International of Gynecology and Obstetrics

P value was calculated with *χ*^2^ test.

*P < 0.05 is considered significant

### Efficacy analysis

**Fig. 2:**
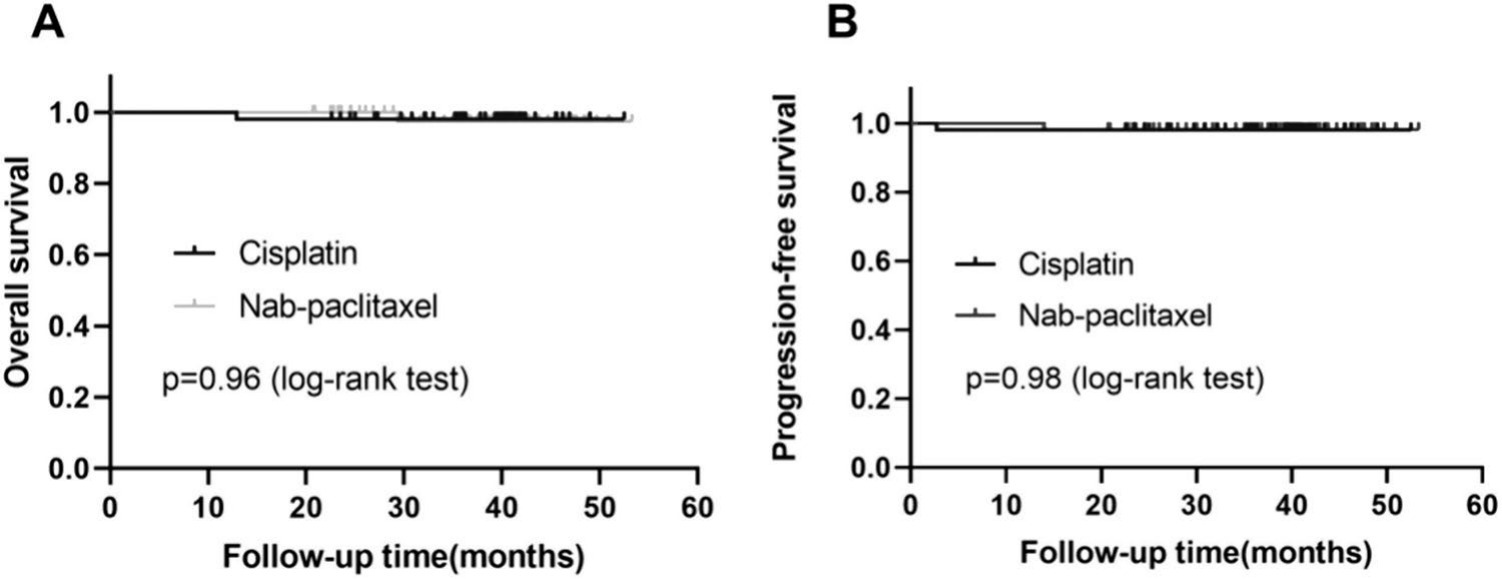
Kaplan–Meier analysis for overall survival and progression-free survival in patients. **A** overall survival (OS) and **B** progression-free survival (PFS)

The median follow-up time was 38.7 months. It did not appear to be a difference between the two groups when it came to overall survival (P = 0.96, HR 0.93, 95% CI 0.058 to 15.06) and progression-free survival (P = 0.98, HR 1.03, 95% CI 0.06 to 16.46, Fig. 2). A nab-paclitaxel patient and a cisplatin patient died during follow-up. The 3-year overall survival was 97.4% in the nab-paclitaxel group and 98.1% in the cisplatin group; and the 3-year progression-free survival was 98.1% and 98.1%, respectively.

## Treatment-related toxicities

Table 2 shows all the adverse events. This study showed that a higher frequency of hematologic toxicity in the cisplatin group than the nab-paclitaxel group (P < 0.05). Among that, twenty-four patients in the cisplatin group had grade 3–4 leukopenia, whereas ten of the patients in the nab-paclitaxel group had leukopenia above grade 3 (P = 0.03). Most of patients suffered grade 1–2 neutropenia, the difference between both groups was statistically significant (P = 0.021). The majority of anemia and thrombocytopenia that occurred in both groups were below grade 3, also, grade 1–2 thrombocytopenia in the cisplatin group were more prevalent than in the nab-paclitaxel group (32.7% vs. 9.5%; P = 0.014). Gastrointestinal reactions, such as vomiting, nausea, and anorexia were significantly reduced in the nab-paclitaxel group compared with those in the cisplatin group (P < 0.05). Regarding the effects on alopecia, the incidence rate of the nab-paclitaxel group was higher than that of the cisplatin group (P = 0.001). In addition, no differences were found between the groups with regard to other adverse reactions. None of the patients died during treatment.

**Table 2:** Adverse events during treatment

Cisplatin group (n = 52)					Nab-paclitaxel group (n = 53)				p value
Adverse event	Grade 1	Grade 2	Grade 3	Grade4	Grade1	Grade2	Grade3	Grade4	
*Hematological*									
Leukopenia	7 (13.5)	21( 40.4)	23 (44.2)	1 (1.9)	17 (32.1)	23 (43.4)	10 (18.9)	0	0.030*
Neutropenia	12 (23.1)	16 (30.8)	13 (25)	1 (1.9)	15 (28.3)	18 (33.9)	6 (11.3)	0	0.021*
Anemia	28 (53.8)	12 (23.1)	2 (3.8)	0	22 (41.5)	8 (15.1)	0	0	0.390
Thrombocytopenia	10 (19.2)	7 (13.5)	0	0	3 (5.7)	2 (3.8)	0	0	0.014*
*Non-hematological*									
vomiting	11 (21.2)	8(15.4)	1 (1.9)	0	6 (11.3)	2 (3.8)	0	0	0.041*
Nausea	12 (23.1)	8 (15.4)	2 (3.8)	0	8 (15.1)	3 (5.7)	0	0	0.038*
Anorexia	13 (25.0)	8 (15.4)	3 (5.8)	0	8 (15.1)	2 (3.8)	1 (3.8)	0	0.035*
Diarrhea	7 (13.5)	3 (5.8)	0	0	8 (15.1)	2 (3.8)	0	0	0.875
Constipation	6 (11.5)	3 (5.8)	0	0	5 (9.4)	3 (5.7)	0	0	0.950
Urocystitis	3 (5.8)	0	0	0	5 (9.4)	0	0	0	0.479
Weight loss	11 (21.2)	2 (3.8)	0	0	10 (18.9)	2 (3.8)	0	0	0.789
Fatigue	8 (15.3)	4 (7.7)	0	0	6 (11.3)	2 (3.8)	0	0	0.545
Hepatotoxic event	4 (7.7)	0	0	0	2 (3.8)	0	0	0	0.387
Alopecia	2 (3.8)	3 (5.8)	0	0	5 (9.4)	17 (32.1)	0	0	0.001*
Peripheral neuropathy	0	0	0	0	2 (3.8)	0	0	0	0.157

Data are shown as median n (%)

As prespecified by protocol, differences in adverse events were analyzed using χ^2^ test

*P < 0.05 is considered significant

### Therapy compliance

Regarding EBRT compliance, the median radiotherapy dose was 50.4 Gy and the median dose per fraction was 1.8 Gy. The dose and duration of radiotherapy were well-balanced between the treatment groups. As seen in Table 1, overall, patients who accepted 4–6 cycles of concurrent chemotherapy, was 46/52 (88.5%) in the cisplatin group and 52/53 (98.1%) in the nab-paclitaxel group, whereas the remaining patients completed 3 cycles. Chemotherapy compliance had significant difference between two groups (P = 0.001).

## Discussion

Following radical hysterectomy, patients with cervical squamous cell carcinoma treated with nab-paclitaxel or cisplatin as adjuvant CCRT had similar 3-year survival rates. Patients in our study with a higher overall survival, partly because most of them received four to six concurrent chemotherapy cycles, a good level of patient compliance was observed. Additionally, the utilization of more precise intensity-modulated radiotherapy (IMRT) technology in recent years may contribute to this phenomenon. To the best of our knowledge, this study represents a potentially groundbreaking retrospective control study, as it demonstrates the comparable efficacy of nab-paclitaxel-based concurrent chemoradiotherapy (CCRT) and standard cisplatin-based CCRT in terms of both progression-free survival and overall survival in the context of postoperative adjuvant therapy for patients with early-stage cervical cancer.

Current guidelines recommend adjuvant radiotherapy with or without chemotherapy for the management of cervical cancer after radical surgery, when postoperative pathological factors including lymphovascular space invasion, stromal invasion, tumor histology (adenocarcinoma component), close or positive surgical margins, and tumor size (cm). Intermediate risk was defined according to Sedlis criteria in GOG protocol 92, it was suggested that significant heterogeneity existed in Sedlis criteria: patients with positive lymphovascular space invasion and deep 1/3 stromal invasion had a higher risk of reccurence and disease-specific death (Sedlis et al. 1999; Cao et al. 2021). For these intermediate-risk patients, adjuvant concurrent chemoradiotherapy was recommended at our institution. For patients with node negative but large primary tumors, deep stromal invasion, and/or lymphovascular space invasion, there is no current consensus on the role of chemotherapy in addition to adjuvant radiotherapy (Kim et al. 2020; Rodriguez et al. 2022). Thus, the current modality for risk-adapted postoperative treatment needs further investigation.  Cisplatin, remains a popular agent that has predominated the chemotherapy of cervical cancer for a long time. In the 1990s, several large prospective randomized trials were conducted, it was stated that cisplatin-based radiochemotherapy decreased the relative risk of recurrence and the mortality (Keys and Morris; Rose 1999). Based on the findings of the trials, NCCN guidelines recommended platinum-based concurrent chemoradiotherapy as the standard therapy for cervical carcinoma, especially cisplatin. However, due to the potential toxicity frequently seen in the gastrointestinal tract and kidney, clinical use of cisplatin may be restricted. In the clinical setting, paclitaxel has been used as a good radiosensitizer in a variety of disease sites including non-small-cell lung cancer, head and neck, cervical, and so on (Liang 2017 and Sugawara 2021; Han 2023; Kim 2006). Chemical solvents such as Cremophor EL and ethanol are used in paclitaxel formulations to improve its solubility. These solvents are known to cause some side effects, including dose-limiting toxicity and acute hypersensitivity reactions. Nab-paclitaxel was a new formulation that was initially developed more than a decade ago, and decreased the incidence of serious toxicities, including severe allergic reactions compared with solvent-based paclitaxel (sb-PTX). Because of polyethoxylated castor oil contained in sb-PTX, premedication with steroids and histamine H-2 blockers is generally required for safety (Gelderblom et al. 2001). It was showed that nab-paclitaxel has a significant antiproliferative effect on cervical cancer Hela cells (Gurses et al. 2013). In a vivo study regarding ovarian or mammary carcinomas, nab-paclitaxel exhibited strong antitumor efficacy against both tumors as a single agent and it improved radiotherapy in a supra-additive manner. It enhanced tumor response in terms of block tumor growth and increase tumor cure rate to irradiation (Wiedenmann et al. 2007). These preclinical findings demonstrated that this novel paclitaxel will be thus a good candidate for testing in clinical chemoradiotherapy trials. To the best of our knowledge, there have been several trials regarding nab-paclitaxel for different cancer types, such as melanoma, head and neck cancer, pancreatic cancer, and ovarian cancer, among others (Oppelt et al. 2021 and Koay et al. 2022; Li et al. 2022; Colombo et al. 2023). It was demonstrated that nab-paclitaxel cross endothelial cell monolayers and enter tumors through endogenous albumin transport pathways, including receptor-mediated transcytosis (Desai et al. 2006 and Desai et al. 2009). In a preclinical study, fourfold more nab-paclitaxel was delivered to tumors than sb-PTX, with faster and deeper tissue penetration and high systemic exposure time is shorter. However, these improved effects were achieved without increased normal tissue toxicity. This lack of modification of normal tissue radioresponses may be attributable to selective accumulation of nab-paclitaxel in tumors (Desai et al. 2006). Consistent with the preclinical data, paclitaxel clearance and volume of distribution were significantly higher for nab-paclitaxel than for sb-PTX in humans patients (Sparreboom et al. 2005). Nab-paclitaxel is well tolerated in women with gynecologic cancer including cervical malignancy, experiencing no reactions or major side effects to the drug (Fader and Rose 2009). The advantages of nab-paclitaxel versus sb-PTX likely contribute to more favorable efficacy and safety profile. This may, in turn, explain the lower frequency of some severe adverse events, such as myelosuppression. Our findings show that the administration of cisplatin brought out more severe hematological toxicities and gastrointestinal reactions than the nab-paclitaxel, which led to the lower completion rate of chemotherapy and radiotherapy suspension.      

In the current study, we observed that nab-paclitaxel presented milder adverse reactions but similar effect with cisplatin. Nab-paclitaxel may be considered a leading candidate for future studies of combinations of agents in both the adjuvant and advanced disease settings, especially evaluating weekly dosing schedules. The recommended doses and schedules of dose density chemotherapy regimen nab-paclitaxel by the NCCN for the systemic treatment of recurrent or metastatic breast cancer is 100 ~ 150 mg/m^2^ qw. It was reported that the recommended dose for nab-paclitaxel was 60 mg/m^2^ weekly when given standard weekly concurrent intensity modulated radiation therapy (IMRT) in patients with head and neck squamous cell carcinoma (Suntharalingam et al. 2012). Jiang et al. found that the MTD of nab-paclitaxel was 50 mg/m^2^ in locally advanced cervical cancer patients undergoing concurrent radiation therapy with nab-paclitaxel plus cisplatin (40 mg/m^2^) weekly (Jiang et al. 2023). In our study, the dose of the nab-paclitaxel we used weekly was 100 mg in CCRT, the body surface area range of nab-paclitaxel group patients was 1.44–1.80 m^2^, which was equal to 55–70 mg/m^2^ dose of nab-paclitaxel. Nab-paclitaxel has three Food and Drug Administration (FDA) approved indications: locally advanced or metastxatic non-small cell lung cancer, metastatic adenocarcinoma of the pancreas, and recurrent metastatic breast cancer (Gradishar et al. 2005; Gong et al. 2017; Von Hoff et al. 2013). The pathological patterns of these cancers are mainly adenocarcinoma, few data concerning nab-paclitaxel against squamous cell carcinoma of any type. Researchers found that nab-paclitaxel show better results in patients with squamous histology because of its antiangiogenic properties (Alberts et al. 2012; Cecco et al. 2014). It is interesting to note that the predominant pathology was squamous cell carcinoma in our trial (89 of 102 patients, 87.3%). Our trial showed that patients undergoing nab-paclitaxel-based CCRT had similar OS and PFS with cisplatin-based CCRT. The relatively small sample size and short follow-up time may limited the value of the survival endpoints. Longer follow-up is needed to fully assess survival and long-term toxic effects. Further investigations are needed.

## Conclusions

The results of our study indicate that nab-paclitaxel-based CCRT may serve as a viable substitute for cisplatin-based CCRT in the treatment of cervical cancer. Given the advantageous properties of nab-paclitaxel, it has the potential to play a crucial role in the future combined therapy of cervical cancer and other malignancies.

## Data Availability

No datasets were generated or analysed during the current study.
